# Ceramide Synthase 1 Inhibits Brain Metastasis of Non-Small Cell Lung Cancer by Interacting with USP14 and Downregulating the PI3K/AKT/mTOR Signaling Pathway

**DOI:** 10.3390/cancers15071994

**Published:** 2023-03-27

**Authors:** Yiquan Xu, Junfan Pan, Ying Lin, Yun Wu, Yusheng Chen, Hongru Li

**Affiliations:** 1 Shengli Clinical Medical College, Fujian Medical University, No. 134 East Street, Fuzhou 350001, China; xuyiquan1018@fjmu.edu.cn (Y.X.); pjf1162482056@163.com (J.P.); linying0323@163.com (Y.L.); wuyun3935@163.com (Y.W.); 2 Department of Respiratory Medicine and Critical Care Medicine, Fujian Provincial Hospital, No. 134 East Street, Fuzhou 350001, China; 3 Fujian Provincial Researching Laboratory of Respiratory Diseases, No. 134 East Street, Fuzhou 350001, China

**Keywords:** ceramide synthase 1, non-small cell lung cancer, brain metastasis, PI3K/AKT/mTOR signaling pathway, ubiquitin-specific protease 14

## Abstract

**Simple Summary:**

Brain metastasis is common in patients with non-small cell lung cancer and is associated with a poor prognosis. Ceramide synthase 1 participates in malignancy development, but its potential role in non-small cell lung cancer brain metastasis remains unclear. Using bioinformatics analysis and molecular biotechnology, we found that ceramide synthase 1 could inhibit non-small cell lung cancer brain metastasis in vivo and in vitro. Mechanistically, ceramide synthase 1 interacted with ubiquitin-specific protease 14 and inhibited brain metastasis progression by downregulating the PI3K/AKT/mTOR signaling pathway. We suggest that ceramide synthase 1 is an effective therapeutic target for non-small cell lung cancer patients with brain metastases.

**Abstract:**

Brain metastasis (BM) is common in patients with non-small cell lung cancer (NSCLC) and is associated with a poor prognosis. Ceramide synthase 1 (CERS1) participates in malignancy development, but its potential role in NSCLC BM remains unclear. This study aimed to explore the physiological effects and molecular mechanism of CERS1 in NSCLC BM. CERS1 expression was evaluated in NSCLC tissues and cell lines, and its physiological roles were subsequently explored in vivo and in vitro. Mass spectrometry and co-immunoprecipitation were performed to explore CERS1-interacting proteins. The associated signaling pathways of CERS1 in NSCLC BM were further investigated using bioinformatics analysis and molecular biotechnology. We demonstrated that CERS1 was significantly downregulated in NSCLC cell lines and BM tissues, and its upregulation was associated with better prognoses. In vitro, CERS1 overexpression inhibited cell migration, invasion, and the ability to penetrate the blood-brain barrier. Moreover, CERS1 interacted with ubiquitin-specific protease 14 (USP14) and inhibited BM progression by downregulating the PI3K/AKT/mTOR signaling pathway. Further, CERS1 expression substantially suppressed BM tumor formation in vivo. This study demonstrated that CERS1 plays a suppressor role in NSCLC BM by interacting with USP14 and downregulating the PI3K/AKT/mTOR signaling pathway, thereby serving as a novel therapeutic target for NSCLC BM.

## 1. Introduction

Lung cancer is the most common cancer type and is the leading cause of cancer-related deaths worldwide [1]. Non-small cell lung cancer (NSCLC) accounts for approximately 85% of all lung cancers [2], with over 25% of patients with NSCLC presenting with brain metastases (BM) at the time of diagnosis [3]. Although various treatments have made great progress, the median overall survival (OS) of NSCLC BM patients is only about 12 months [4]. Therefore, there is an urgent need to identify the underlying molecular mechanisms of BM in NSCLC and develop new targeted therapies.

BM development in NSCLC is a very complicated process, with recent studies revealing key molecules and mechanisms. Genetic analyses have demonstrated that NSCLC patients with an epidermal growth factor receptor mutation or anaplastic lymphoma kinase rearrangement are at greater risk of brain dissemination [5,6]. Molecular analyses have revealed that amplified MYC, YAP1, and MMP13 contribute to BM formation in NSCLC [7]. In addition, activation of the WNT/TCF pathway has been identified as a determinant of BM in lung adenocarcinoma, acting through transcription factors LEF1 and HOXB9 to enhance the competence of tumor cell invasion and proliferation [8]. Despite extensive research on the key molecules of NSCLC BM, the underlying mechanism remains unclear.

Ceramide is the core component of biologically active sphingolipids [9], and abnormal levels have been detected in several diseases [10,11,12]. Ceramide is primarily produced via the catabolic pathway, in which sphingosine is re-acylated by ceramide synthase (CERS) in the lysosome [13]. In this process, each CERS produces ceramide with a specific acyl chain length, which endows cells with specific ceramide properties, inhibiting or promoting cell proliferation [14,15,16]. Notably, abnormal CERS expression was significantly correlated with the pathogenesis of various cancers. For example, the CERS1 expression level in head and neck tumors is lower than that in normal tissues and is associated with lymphatic infiltration and lymph node metastasis [17]. CERS2 overexpression inhibits breast cancer cell invasion by decreasing MMP-2 and MMP-9 activity and inhibiting extracellular matrix degradation [18]. Further, Chen et al. [19] reported that CERS4 was highly expressed in liver cancer tissues and promoted liver cancer cell proliferation through the NF-κB signaling pathway. CERS6 has been identified as a cancer-promoting factor for lung, colon, breast, and ovarian cancers as well as other malignant tumors [20,21,22]. Nevertheless, the physiological effects and molecular mechanism of CERS in NSCLC BM, especially CERS1, have not been reported.

In this study, we aimed to evaluate the expression of CERS (CERS1–6) in NSCLC tissues and cell lines. We found that CERS1 was significantly associated with NSCLC BM. We further investigated the physiological roles and underlying mechanisms of CERS1 in NSCLC BM in vivo and in vitro.

## 2. Materials and Methods

### 2.1. Study Population and Clinical Data Collection

Cohort 1, which included 28 fresh NSCLC tissues and 19 fresh NSCLC BM tissues, was collected between January 2018 and December 2019 to test CERS1 mRNA expression. Patients with other cancers and tumors of unknown origin were excluded from the study. All tissue pieces were sectioned into approximately 1 cm^3^ by a specialist within 30 min of harvest. The tissues were then rinsed with saline, frozen in liquid nitrogen, and stored at −80℃. Patients with NSCLC were diagnosed via histopathological examinations, and BM was confirmed using magnetic resonance imaging. None of the patients had received radiotherapy, chemotherapy, targeted therapy, or other biological treatments before surgery.

Additionally, we retrospectively collected 71 paraffin-embedded sections of NSCLC specimens as cohort 2, which included 31 tissues from patients with NSCLC without BM (BM− group) and 40 tissues from patients with NSCLC BM (BM+ group). Meanwhile, the BM+ group included 28 BM tissues and 12 lung tissues. All the samples were obtained from the Department of Thoracic Surgery and Neurosurgery of Fujian Provincial Hospital between February 2010 and March 2018. Cohort 2 was used to test the CERS1 expression.

We collected clinical and genetic data from the participants and conducted follow-up through telephone calls or outpatient appointments every 3 months. The Tumor Node Metastasis (TNM) stage of NSCLC was defined according to the 7th edition of the American Joint Committee on Cancer TNM staging system. Written informed consent for tissue collection, clinical data analysis, and paper publication was obtained from all participants. This study was approved by the Ethical Review Committee of Fujian Provincial Hospital (K2019-01-052).

### 2.2. Bioinformatics Analysis

The Cancer Genome Atlas (TCGA) (http://cancergenome.nih.gov/, accessed on 1 June 2019) and Genotype-Tissue Expression (GTEx) (https://www.gtexportal.org/, accessed on 1 June 2019) databases used to download RNA-sequencing data from 594 and 288 tissues, respectively, of which 535 were from lung adenocarcinomas and 347 from normal cases. Additionally, the clinical data, including sex, age, race, smoking history, TNM stage, and survival time, were also collected. The publicly available data used in this study met official TCGA data requirements. The differential expression of CERS1 in LUAD tissues and the adjacent normal tissues was analyzed by the “Limma” package. Gene Set Enrichment Analysis (GSEA) was performed in the lung adenocarcinoma (LUAD) cohort to gain insight into the biological pathways in the high- and low-risk subgroups, as defined by CERS1 expression. GSEA was used to search for C2 curated gene sets, C4 computational gene sets, C6 oncogenic signatures, and Kyoto Encyclopedia of Genes and Genomes (KEGG) pathway-curated gene sets from the Broad Institute Molecular Signature Database. Core genes with *p* < 0.01 and a false discovery rate (FDR) < 0.05 were considered statistically significant [23]. The Cancer Cell Line Encyclopedia (CCLE) database (https://portals.broadinstitute.org/ccle, accessed on 15 June 2019) and used to analyze the CERS1 mRNA expression in 28 malignant tumor cells.

### 2.3. Cell Lines and Reagents

A549 cells and the normal human bronchial epithelial cell line BEAS-2B were donated by Professor Huang Yi (Fujian Provincial Hospital). H292, PC-9, and H1299 cell lines were purchased from the Cell Room (School of Medicine, Central South University), and SCC210011 was donated by Dr. Xu Chen (Hong Kong University). 293T cells were purchased from the American Type Culture Collection (Manassas, VA, USA). Human umbilical vein endothelial cell lines (HUVECs) and Human astrocytes (HAs) were purchased from ScienCell (San Diego, CA, USA). HUVECs were cultured in endothelial cell medium (ECM, ScienCell) supplemented with endothelial cell growth factors and 5% fetal bovine serum (FBS). HAs were cultured in astrocyte medium (AM, ScienCell) supplemented with astrocyte growth factors and 2% FBS. Other cells were cultured in RPMI 1640 medium (Gibco^TM^, Waltham, MA, USA) supplemented with 10% FBS. All cells were cultured at 37 °C in a humidified atmosphere of 5% CO_2_. LY294002 is a specific inhibitor of class I PI3K, and the concentration of the treatment was 20 μM.

### 2.4. Quantitative Reverse Transcription-Polymerase Chain Reaction (RT-qPCR)

TRIzol^TM^ reagent (Invitrogen, Waltham, MA, USA) was used to isolate total RNA from NSCLC tumor tissues and cells. Reverse transcription was performed using the PrimeScript™ RT Reagent Kit (Takara, Japan), and real-time fluorescent quantitative PCR was performed using the GoTaq® qPCR Master Mix (Promega, Madison, WI, USA). The 2^−ΔΔCT^ method was used to detect the CERS1 expression level in each sample. GAPDH expression was used as an internal control. The primer sequences used in this study are listed in Table S1.

### 2.5. Establishment of Stable NSCLC Cell Lines

CERS1 lentivirus overexpression and control vectors, lentiviral CERS1 shRNA, as well as the negative control were purchased from Shanghai GeneChem Co., Ltd. (Shanghai, China). H1299 or PC-9 cells were seeded into 24-well plates and then transduced with 1 × 10^8^ TU/mL lentivirus. Cells were cultured at 37 °C and 5% CO_2_ for 24 h. Following a medium change, they were cultured for another 48 h. The stable cell lines were selected by puromycin and used for all in vitro and in vivo experiments. The shRNA-construct sequences and primers designed for full-length CERS1 PCR are shown in Tables S2 and S3, respectively.

Stable expression of firefly luciferase (luc) in the PC-9 cell line was established. Briefly, the PC-9 cells were transfected with the lentiviral vector plasmid pASLenti-pA-Luc2-CMV-EF1-mCherry-P2A-Puro-WPRE, which expresses luc. Then, stable PC-9 cell lines were selected using puromycin. The Bright-Glo Luciferase Assay System (Promega) was used to measure luciferase activity and further confirm the expression of luc.

### 2.6. Western Blotting and Immunoprecipitation

Cell precipitation was supplemented with radioimmunoprecipitation lysis buffer (Beyotime, Shanghai, China), a protease inhibitor cocktail (Beyotime), and phenylmethyl sulfonyl fluoride (PMSF). The protein samples were isolated by SDS-PAGE and transferred to polyvinylidene fluoride membranes (Millipore, Burllington, MA, USA). The membrane was then incubated with the primary antibody and bound to the secondary antibody. Finally, the imprints were visualized using an ECL chemiluminescent reagent (Meilunbio, Dalian, China), and Image Lab Software was used for data analysis. The primary antibodies were as follows: β-Tubulin (YM3030, ImmunoWay, Suzhou, China), CERS1 (sc-293497, Santa Cruz Biotechnology, CA, USA), PI3 Kinase p85 (19H8, Cell Signaling Technology, MA, USA), AKT1 + AKT2 + AKT3 (ab179463, Abcam, Cambridge, UK), AKT1 ^S473^ (ab81283, Abcam), mTOR (ab32028, Abcam), mTOR ^S2448^ (ab109268, Abcam), ZO-1 (ab96587, Abcam), Occludin (ab216327, Abcam), Claudin 5 (ab131259, Abcam), Cleaved Caspase-3 (ab32042, Abcam), Caspase-9 (ab202068, Abcam), Bax (ab32503, Abcam), Bcl-2 (ab32124, Abcam), USP14 (ab235960, Abcam), and MMP-9 (10375-2-AP, Proteintech, Chicago, IL, USA).

Immunoprecipitation (IP) was used to investigate the interaction between endogenous CERS1 and ubiquitin-specific protease 14 (USP14). After PC-9 cells reached 90% confluence, they were washed three times with pre-cooled phosphate-buffered saline (PBS) and dissolved in IP lysis buffer (containing 8 μL PMSF and 8 μL phosphatase inhibitor). The lysate was incubated with anti-CERS1 antibody (sc-293497, Santa Cruz Biotechnology) and anti-USP14 antibody (sc-515812, Santa Cruz Biotechnology) at 4 °C overnight, following which protein A/G Sepharose® beads were added and incubated overnight. The agarose beads were collected and rinsed three times with a lysis buffer, after which the precipitated protein was eluted, denatured in 5× SDS loading buffer, and analyzed using western blotting.

### 2.7. Immunohistochemistry (IHC) Staining

IHC staining was performed on a formalin-fixed, paraffin-embedded patient tissue sample. Protein expression of CERS1 and MMP9 was detected. The following antibodies were used: CERS1 (AB198799, Abcam) and MMP9 (10375-2-AP, Abcam) were used. Briefly, tissue sections were incubated with rabbit anti-CERS1 and anti-MMP9 antibodies overnight at 4 °C. Normal goat serum was used as a negative control. After washing, tissue sections were incubated with biotinylated anti-rabbit secondary antibodies (Santa Cruz Biotechnology). Following further incubation with the streptavidin-horseradish peroxidase complex (Sigma, St. Louis, MO, USA), sections were immersed in 3,3-diaminobenzidine, counterstained with 10% Mayer’s hematoxylin, dehydrated, and mounted. Tissue IHC was scored according to the percentage of positively stained tumor cells and staining intensity. Immunopositivity was independently evaluated by two experienced pathologists.

### 2.8. Immunofluorescence

For immunofluorescence analysis, H1299 and PC-9 cells were seeded onto glass slides and cultured for 24 h. They were then washed three times with PBS and fixed with 4% paraformaldehyde for 10 min. Subsequently, they were washed another three times with PBS, permeabilized with 0.5% Triton X-100, and blocked with 5% bovine serum albumin in PBS for 1 h. Further, they were sequentially incubated with primary and secondary antibodies. Finally, the nuclei were DAPI-stained, and images were captured using a fluorescence microscope.

### 2.9. Cell Counting Kit-8 (CCK8) Assay

Cells were seeded in 96-well plates (5 × 10^3^ cells/well), and proliferation was assessed using CCK-8 (Meilunbio, Dalian, China), according to the manufacturer’s protocol. Briefly, 10 μL of CCK-8 solution was added to the culture medium, plates were incubated for 2 h at 37 °C in 5% CO_2_, and the absorbance was measured at 450 nm. Cell proliferation was detected on days 0, 1, 2, 3, and 4. All experiments were repeated at least three times.

### 2.10. Flow Cytometry of Cell Cycle

Cells were digested and washed by PBS three times. Then, propidium iodide (PI) (Meilunbio) was used to stain the cells, and they were incubated at 37 °C for 30 min. After incubation, the cells were suspended and analyzed using an Accuri C6 flow cytometer. A ModFit LT program was performed to analyze the data. All experiments were repeated at least three times.

### 2.11. Transwell Migration and Invasion Assays

Transwell chambers (8 μm pore size, BD, Biosciences) were used to conduct cell migration and invasion experiments. For the migration assay, 2 × 10^4^ cells were diluted in 200 μL serum-free medium and placed in the upper chamber. For the invasion assay, 5 × 10^5^ cells were transferred to the upper chamber coated with Matrigel (Corning, NY, USA). The lower chamber contained 600 μL of medium with 15% FBS as the chemical attractant. Following 24 h of incubation, the upper chamber was removed, and the cells attached to the lower membrane surface were fixed with methanol and stained with 5% crystal violet (Sigma, USA). An IX71 inverted microscope (Olympus, Tokyo, Japan) was used to image and count the cells. All experiments were repeated three times.

### 2.12. Wound-Healing Assay

Cells were seeded into 6-well plates (1 × 10^5^ cells/well). Once confluent, the monolayer was scratched with a 10 μL plastic pipette tip to form a uniform wound. Further, the monolayer was washed with PBS, and cells were cultured in the medium without FBS. The distance between the two edges of the migrating cell sheet was determined using a phase-contrast microscope. All experiments were repeated at least three times.

### 2.13. Construction of a Blood-Brain Barrier Model (BBB)

HUVECs and HAs were co-cultured on opposite sides of a 24-well transwell polycarbonate insert (3 μm pore size, Coring) to develop the in vitro BBB model [24]. The transwell insert was coated with 2% gelatin (Sigma, USA) for 45 min and placed upside-down. Then, 1 × 10^5^ HAs were plated on the underside of the insert. The cells were incubating at 37 °C in 5% CO_2_ and being fed with astrocyte medium every 15–30 min. After 4 h, the inserts were turned over and placed into 24-well plates. Astrocyte medium (1 mL) was added to the lower chamber, and HAs were incubated for an additional 24 h. Further, the upper chamber of the inserts was seeded with 5 × 10^4^ HUVECs and incubated for 3 d. The permeability of the BBB model was measured by tight junction-related protein expression and horseradish peroxidase (HRP) flux. For HRP analysis, the upper chamber received 1 mL of RPMI 1640 medium (without red phenol), which was supplemented with 50 μg/mL HRP. Next, 1.5 mL of culture medium was added to the lower chamber. An amount of 50 μL of the culture medium was removed from the lower chambers, and 100 μL 3,3′,5,5′-tetramethylbenzidine was added at the indicated times (5, 15, 30, 60, and 120 min). Then, the medium was incubated at 25 °C for 30 min, and the reaction was stopped with 1 M of H_2_SO_4_. The absorbance of the medium was measured at 450 nm, and permeability was calculated according to the following formula: PHRP% = (CHRP lower chamber × VHRP lower chamber)/(CHRP upper chamber × VHRP upper chamber) × 100%.

After constructing the BBB model, 1 mL of 1 × 10^5^/mL cells expressing EGFP were added to the upper chamber. The PBS was used to wash the cells in the lower chamber after 24 h. The cells were fixed with 4% paraformaldehyde at room temperature for 20 min. Finally, the cells were imaged under a fluorescence microscope. The average number of EGFP-migrated cells was counted in five random fields.

### 2.14. Mass Spectrometry (MS) Analysis

Briefly, liquid chromatography tandem mass spectrometry (LC-MS/MS) was carried out using a Q-Exactive mass spectrometer coupled with an Easy nLC (Thermo Fisher Scientific, Waltham, MA, USA). The peptide sample was loaded onto the C18-reversed phase analytical column (50 μm × 15 cm) in buffer A (0.1% formic acid in high-performance liquid chromatography grade water) and separated with buffer B (80% acetonitrile and 0.1% formic acid) at a flow rate of 300 nL/min. Further, the peptide was loaded into the Q Exactive mass spectrometer. MS analysis was set for 60 min in positive ion mode. MS data were acquired using the data-dependent top 10 most abundant precursor ions from the full scan (350–1800 *m*/*z*) for higher-energy C-trap dissociated fragmentation. Technical triplicates were performed to identify the relevant proteins. The criterion for the positive proteins was for the peptide scores of specific peptides to reach a significant threshold of FDR = 0.01.

### 2.15. Subcutaneous Xenograft Model

PC-9 cells (5 × 10^6^ cells/point) were resuspended in 100 μL serum-free medium and inoculated onto the subcutaneous region of female BALB/c nude mice (4–5 weeks old) (Wssydw, Fuzhou, China). The tumor was monitored and measured every 3 d. We calculated the volume of the tumor as follows: 0.5 × tumor length × tumor width^2^. Experimental endpoints were tumor sizes exceeding 2 cm or the development of further complications affecting animal welfare. Mice were humanely euthanized, and the tumors were harvested for weighing, measuring, and further studying. All animal experiments were approved by the Institutional Animal Care and Use Committee of Fujian University of Traditional Chinese Medicine (FJTCM IACUC 2021188).

### 2.16. Orthotopic Xenograft Model

For the orthotopic xenograft model, mice were first anesthetized, after which a 1 mm hole was drilled in the skulls using a syringe needle. Further, 5 × 10^6^ PC-9 cells, which stable express luc, were resuspended in 10 μL serum-free medium and slowly injected into the mice’s brains. Body weight was measured every 3 days following the operation. On week 6, some mice appeared to have clinical symptoms of BM, including immobility, weight loss, or a hunchback. Subsequently, bioluminescence imaging was performed. Each mouse was intraperitoneally injected with 150 mg/kg luciferin (PerkinElmer, Waltham, MA, USA) for 10 min and then imaged using the IVIS Lumina In Vivo imaging system (Calipers, Hopkinton, MA, USA). The brain was excised, fixed with 10% formalin, and sectioned into 2–3 mm sections. Hematoxylin and eosin staining was used to observe the location and number of brain metastases.

### 2.17. Statistical Analysis

TCGA and GTEx data were analyzed using the “Limma” package of R software (version 4.0.5), and all other experimental results were analyzed using SPSS (version 20.0, IBM, Inc., Armonk, NY, USA). The unpaired *t* test was performed for comparisons between the two groups, and the χ^2^ test was conducted to investigate discrepancies in distribution between the two groups. A one-way ANOVA with a post-hoc Tukey HSD test was used to determine the differences between multiple groups. The Kaplan–Meier test was conducted to compare survival differences among groups. The results were reported as the mean ± standard deviation. Statistical significance was set at *p* < 0.05, * *p* < 0.05, ** *p* < 0.01, and *** *p* < 0.001.

## 3. Results

### 3.1. Low CERS1 Expression in NSCLC BM Tissues Is Associated with Better Prognoses

**Figure 1:**
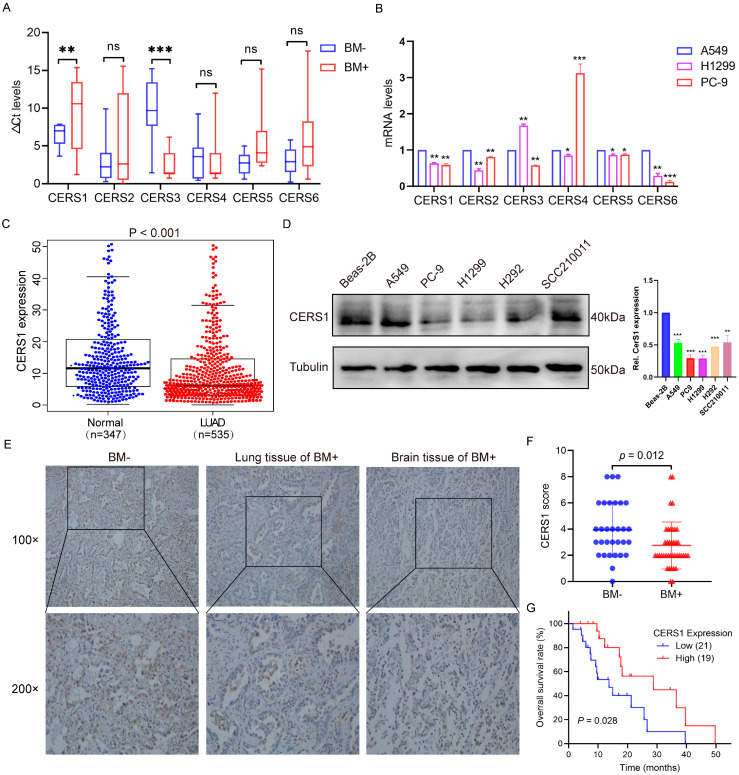
CERS1 exhibits low expression in patients with NSCLC BM. (**A**) mRNA levels of CERS1-6 in fresh NSCLC and BM tissues. (**B**) mRNA expression of CERS1-6 in NSCLC cell lines with different BM tendencies. (**C**) CERS1 expression in lung adenocarcinoma and normal tissues based on TCGA and GTEx database analyses. (**D**) CERS1 expression in NSCLC cell lines was tested via western blotting. (**E**) Representative immunohistochemical staining images of CERS1 in lung tissues without BM (BM−), lung tissue of BM+, and brain tissue of BM+. (**F**) A summary of immunohistochemical staining results for CERS1 expression. (**G**) Kaplan–Meier plotted the overall survival of patients stratified by CERS1 expression. ns, no-significant; * *p* < 0.05; ** *p* < 0.01; *** *p* < 0.001.

To identify the key ceramide genes associated with NSCLC BM, we used RT-qPCR to detect CERS1–6 expression in 47 fresh tissues (28 NSCLC and 19 BM) of cohort 1, and NSCLC cells with different tendencies for BM. We observed that CERS1 expression was significantly lower in BM tissues than in NSCLC tissues (*p* < 0.01, Figure 1A). Additionally, CERS1 showed a lower expression in cells with a high tendency for BM, including H1299 and PC-9 lines, compared to A549 (*p* < 0.01, Figure 1B). The five other CERS types did not show the same expression trend as CERS1. Furthermore, CERS1 was generally downregulated in various cancer cells, according to the CCLE database (Figure S1). Based on the TCGA and GTEx database analyses, CERS1 had decreased expression in LUAD tissues compared to normal tissues (*p* < 0.001, Figure 1C). CERS1 protein expression in most NSCLC cell lines was significantly lower than in BEAS-2B cells (Figure 1D).

Furthermore, we performed IHC staining to detect the pathological effect of CERS1 in 71 NSCLC tissues from cohort 2. As shown in Figure 1E,F, CERS1 was mainly located in the cytoplasm and weakly expressed in the BM^+^ group compared to the BM^−^ group (*p* = 0.012). Kaplan-Meier analysis revealed that patients with BM showing high CERS1 expression exhibited higher tissue differentiation (Table 1) and had a significantly longer OS than those with low CERS1 expression (*p* = 0.028, Figure 1G). These results indicated that CERS1 may play a tumor suppressor role and that elevated CERS1 levels predict better prognoses in patients with NSCLC BM.

### 3.2. CERS1 Inhibits Tumorigenesis and BM of NSCLC In Vitro

**Figure 2:**
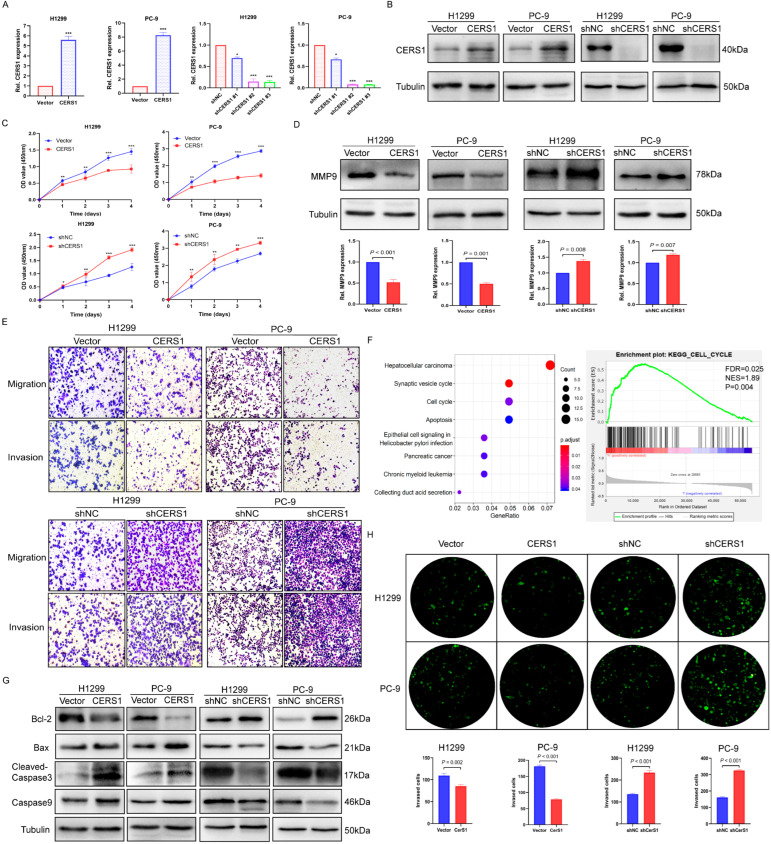
CERS1 overexpression inhibits the proliferation, migration, and invasion of NSCLC cells. (**A**,**B**) The efficiency of CERS1 overexpression and depletion was analyzed using RT-qPCR and western blotting. (**C**) The ability of H1299 and PC-9 cells to proliferate following CERS1 transfection was tested using CCK-8 assays. (**D**) MMP-9 expression was evaluated using western blotting. (**E**) The ability of H1299 and PC-9 cells to migrate and invade was tested through transwell assays. (**F**) KEGG and GSEA analyses were used to determine the signaling pathway associated with CERS1. (**G**) The impact of CERS1 on cell apoptosis was detected using western blotting. (**H**) Effect of CERS1 on the ability of lung cancer cells to penetrate the BBB model. * *p* < 0.05; ** *p* < 0.01; *** *p* < 0.001.

To investigate the physiological role of CERS1 in NSCLC cells, we used PC-9 and H1299 cells to establish stable CERS1 overexpression and knockdown (shCERS1) cell lines. RT-qPCR and western blotting were conducted to confirm the efficiency of CERS1 overexpression and depletion (*p* < 0.05, Figure 2A,B). We then explored the proliferative and metastatic effect of CERS1 on NSCLC cells. CCK-8 assays revealed that CERS1 overexpression significantly inhibited NSCLC cell proliferation, while shCERS1 exhibited the opposite effect (Figure 2C). As MMP-9 expression was linked to metastatic potential, we used western blotting to detect the relationship between CERS1 and MMP-9. The results revealed that CERS1 expression significantly decreased MMP-9 protein levels (*p* < 0.01, Figure 2D). Wound-healing and transwell assays revealed that CERS1 overexpression reduced NSCLC cells invasion and metastasis, while CERS1 knockdown had the opposite effect (Figure 2E and Figure S2A).

KEGG and GSEA enrichment analyses showed that CERS1 was closely associated with the cell cycle and apoptosis (*p* < 0.05, Figure 2F). Using Annexin V-APC, Western blotting, and cell cycle assays, we found that CERS1 overexpression increased the proportion of apoptotic cells and enhanced the expression of apoptosis-related proteins, including Bax, cleaved-Caspase3, and Caspase9. In contrast, CERS1 knockdown produced the opposite result (Figure 2G), confirming that CERS1 expression promotes NSCLC cell apoptosis. In addition, flow cytometry showed that CERS1 overexpression can block cells in the G1/S phase, confirming that CERS1 participates in cell cycle regulation (*p* < 0.05, Figure S2B).

To explore the impact of CERS1 on NSCLC BM cells, we constructed an in vitro BBB model. HRP flux and the expression of tight junction-related proteins were used to evaluate the BBB permeability. The results showed that the HRP permeability was lowest when HUVECs and HAs were cocultured for 72 h (Figure S3A). When HUVECs were incubated for 72 h, the tight junction proteins ZO-1, Occludin, and Claudin-5 had the highest expression (Figure S3B). These results suggest that the BBB model could achieve an ideal tight junction state and show the lowest permeability when cells were cocultured for 72 h. We then incubated cell lines that were stably transduced with the CERS1 lentivirus and used the constructed BBB model. Our results showed that the number of NSCLC cells that crossed the BBB model decreased when CERS1 was overexpressed, while CERS1 knockdown caused the opposite effect (Figure 2H). These results indicate that CERS1 may inhibit NSCLC BM in vitro.

### 3.3. CERS1 Functions by Interacting with USP 14 Protein

**Figure 3:**
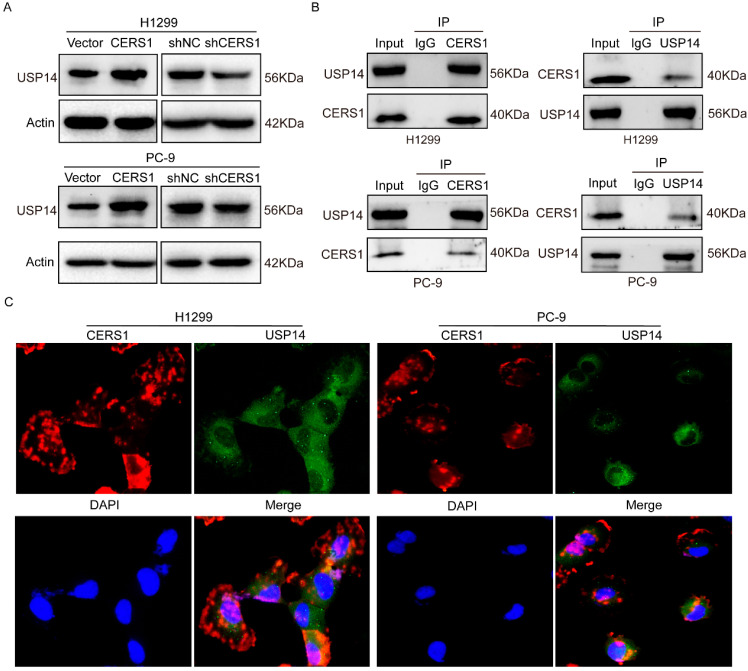
Interaction between CERS1 and transcription factor USP14. (**A**) Expression of indicated proteins was evaluated using western blotting. (**A**) USP14 expression following CERS1 knockdown was detected using western blotting. (**B**) Co-IP demonstrated an interaction between CERS1 and USP14. (**C**) Immunofluorescence was performed to detect CERS1 and USP14 localization in cells.

To explore the potential interacting proteins of CERS1 involved in NSCLC BM, we performed LC-MS/MS analysis on CERS1 pull-down samples. The results identified 1642 CERS1-binding proteins, according to the criterion of peptide scores, to have an FDR = 0.01 (Figure S4). Among the top positive proteins, USP14 was found to be closely related to malignant tumor development [25,26]. Therefore, we aimed to detect the relationship between CERS1 and USP14. As shown in Figure 3A, decreased USP 14 protein expression was observed with CERS1 knockdown. In addition, Co-IP assays revealed an interaction between CERS1 and USP14 (Figure 3B) and that they were co-localized in the cytoplasm (Figure 3C). These results suggest that CERS1 interacts with USP14, which may further impact biological functions.

### 3.4. CERS1 Overexpression Downregulates the PI3K/AKT/mTOR Signaling Pathway

**Figure 4:**
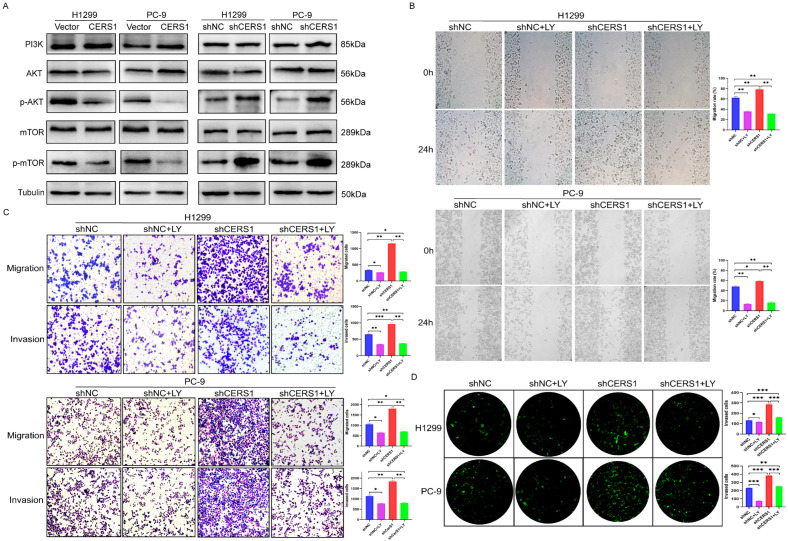
CERS1 expression attenuates the PI3K/AKT/mTOR signaling pathway. (**A**) Expression of indicated proteins was evaluated using western blotting. (**B**,**C**) The effect of LY294002 treatment on the proliferation, migration, and invasion abilities of NSCLC cells stably transfected with shCERS1 was tested via wound-healing and transwell assays. (**D**) After adding LY294002, the ability of CERS1 knockdown cells to cross the BBB was assessed. * *p* < 0.05; ** *p* < 0.01; *** *p* < 0.001.

Furthermore, we investigated the molecular mechanism by which CERS1 overexpression inhibits NSCLC cell growth. We found that CERS1 is closely associated with PI3K and AKT in the apoptosis signaling pathway (Figure S5). In addition, USP14 is known to regulate the PI3K/AKT pathway and affect the malignant phenotype of tumor cells [27,28]. Therefore, we investigated the effect of CERS1 overexpression or knockdown on the PI3K/AKT/mTOR signaling pathway through western blotting. Notably, CERS1 overexpression did not change the total protein levels of AKT and mTOR. However, a decrease in the p-AKT and p-mTOR levels was observed. The opposite results were observed with CERS1 knockdown (*p* < 0.05, Figure 4A). To further explore whether CERS1 caused changes in the biological functioning of NSCLC cells dependent on the PI3K/AKT/mTOR signaling pathway, the cells were cultured for 24 h and then treated with 20 μM LY294002 for another 24 h. The wound-healing and transwell assays showed that LY294002 treatment reversed CERS1 knockdown-induced cell migration and invasion (*p* < 0.05, Figure 4B,C). Furthermore, LY294002 treatment reduced the number of cells passing through the BBB induced by CERS1 knockdown (Figure 4D). Notably, these results indicated that CERS1 downregulated the BM activity of NSCLC cells via the PI3K/AKT/mTOR signaling pathway in vitro.

### 3.5. CERS1 Suppresses Tumorigenesis and BM In Vivo

**Figure 5:**
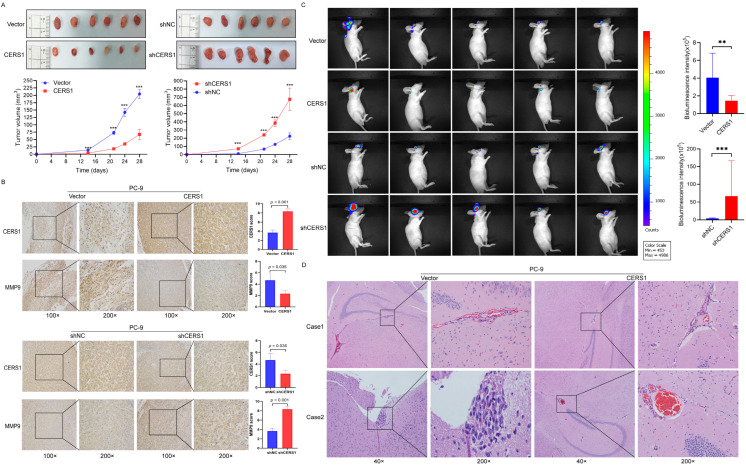
CERS1 suppresses tumorigenesis and brain metastasis in vivo. (**A**) PC-9 cells with stable knockdown and overexpression of CERS1 were used to establish a subcutaneous xenograft model, and the tumor growth curves were plotted. (**B**) Expression of CERS1 and MMP9 in subcutaneously transplanted tumors was detected through immunohistochemical staining. (**C**) Bioluminescent images of mice injected with CERS1-overexpressing cells in the orthotopic xenograft model. (**D**) Hematoxylin and eosin-stained images (40× and 200×) of mice with brain metastases. ** *p* < 0.01; *** *p* < 0.001.

To further study the effect of CERS1 on NSCLC tumorigenesis and BM in vivo, we used PC-9 cells with a stable knockdown or overexpression of CERS1 to establish subcutaneous and orthotopic xenograft implantation models. In the subcutaneous xenograft model, tumor size in the CERS1 overexpression group was smaller than that of the control group (*p* < 0.001, Figure 5A left panel), while the CERS1 knockdown group showed the opposite result (*p* < 0.001, Figure 5A right panel). To explore the tumor characteristics in the subcutaneous xenograft model, we conducted IHC staining for CERS1 and the metastasis-associated marker MMP-9 in xenograft tissues. The results showed that CERS1 expression was significantly higher in the CERS1 overexpression group than in the control group (*p* < 0.001, Figure 5B), while MMP9 showed a reverse trend (*p* = 0.035). Coincidentally, the CERS1 knockdown group showed the opposite result. This suggests that CERS1 expression can significantly inhibit the tumorigenic ability of NSCLC cells in nude mice. Finally, we investigated the effect of CERS1 on BM. Using the orthotopic xenograft implantation model, our findings revealed that mice with CERS1 knockdown had larger BM lesions compared with the control group (*p* < 0.01, Figure 5C,D), while mice with CERS1 overexpression showed the opposite result (*p* <0.001). Collectively, these results indicate that CERS1 suppresses the tumorigenesis and BM of NSCLC in vivo.

## 4. Discussion

**Figure 6:**
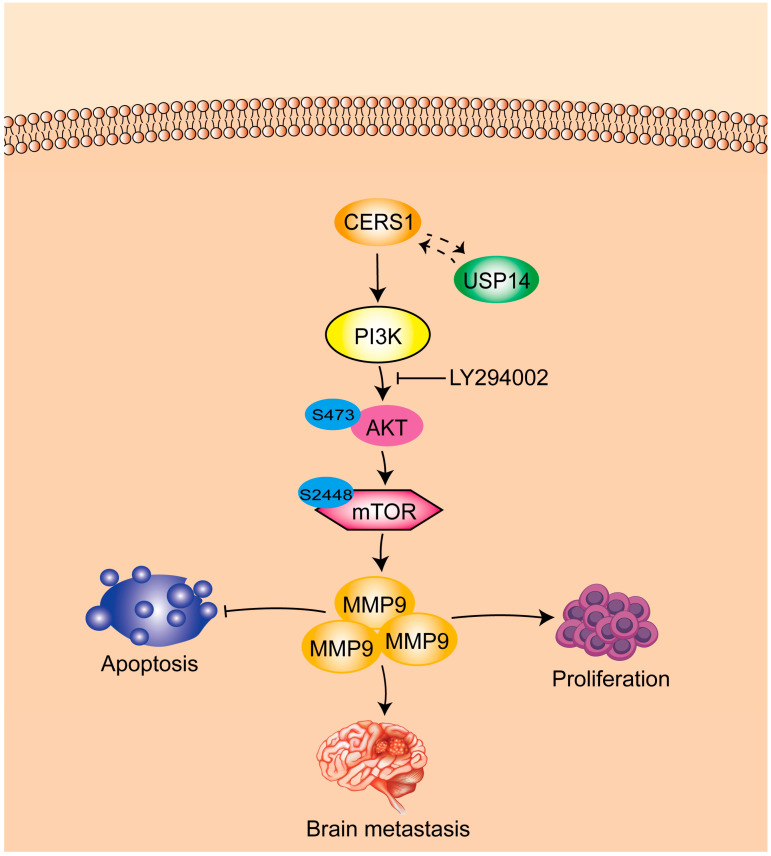
Schematic diagram showing the molecular mechanism of CERS1 in NSCLC BM.

Due to the poor prognosis of patients with NSCLC BM, it is crucial to elucidate the molecular mechanisms underlying this disease to guide the development of diagnostic biomarkers and targeted therapy. In this study, we demonstrated that CERS1 expression was downregulated in patients with NSCLC BM, which suppressed cell proliferation, invasion, migration, and penetration of the BBB. We further demonstrated that the negative regulation of CERS1 was achieved by interacting with USP14 and downregulating the PI3K/AKT/mTOR signaling pathway (Figure 6). To the best of our knowledge, this is the first study describing the role and mechanism of CERS1 in NSCLC BM.

Ceramide is essential to sphingomyelin metabolism and the synthesis of complex sphingolipids [29]. Ceramide metabolism is a complex process, in which each CERS produces a ceramide with a specific acyl chain length. For example, CERS1 and CERS4 mainly produce C18-ceramide, while CERS5 and CERS6 primarily produce C16-ceramide. Ceramides with different acyl chain lengths have different biological functions [30]. As an important member of the CERS family, the role of CERS1 in tumors has attracted increasing attention in recent years. A previous study demonstrated that CERS1 overexpression increased C18-ceramide production in human head and neck squamous cell carcinoma, thereby causing telomerase activity disorder and mitochondrial dysfunction, ultimately leading to cell apoptosis [31]. Wang et al. [32] confirmed that the concentration of C18-ceramide synthesized by CERS1 was significantly lower in glioma tissues than in normal tissues and that CERS1 overexpression in glioma cells could inhibit cell proliferation and promote cell death by activating the endoplasmic reticulum stress response. However, the role of CERS1 in NSCLC BM has not yet been elucidated. In this study, we found that among CERS1–6, CERS1 was expressed at low levels in NSCLC BM tissues, exhibited higher tissue differentiation, and was associated with a better prognosis. Higher tissue differentiation predicted lower malignancy and less invasiveness [33], suggesting that CERS1 expression was closely associated with the lower ability of NSCLC BM. Furthermore, CERS1 negatively regulated cell proliferation, invasion, migration, and penetration of the BBB but promoted NSCLC cell apoptosis. These results are consistent with previously published studies evidencing that CERS1 is a tumor suppressor that plays an important role in cell apoptosis. Although we have detected CERS1 expression in human samples and verified its function in vivo and in vitro, there is currently a lack of drugs corresponding to CERS1 and clinical trials to further confirm the role of CERS1 in NSCLC BM.

USP14, an important member of the USP protein family, participates in a variety of cellular signaling pathways, such as Wnt/β catenin and MAPK/NF-κB [34,35]. Previous studies have revealed that the biological functions of USP14 are regulated by its proteasome and post-modifications [36,37]. USP14 disorders lead to several pathological conditions, including viral infections, hepatosteatosis, neuroglial diseases, and cancers. For example, USP14 overexpression stimulates liver triglyceride accumulation by stabilizing fatty acid synthase [38]. The deubiquitination activity of USP14 induces proliferation, invasion, migration, and vascular mimicry of hepatocellular carcinoma via maintenance of HIF1-α stability [25]. In this study, we demonstrated that CERS1 knockdown negatively regulates USP14 expression. Furthermore, CERS1 and USP14 can form an immune complex and co-localize in cytoplasm. This indicates that CERS1 inhibits NSCLC BM by interacting with USP14.

CERS1 reportedly promotes the killing effect of cisplatin on tumor cells by activating the P38 MAPK signaling pathway [39]. Nevertheless, the mechanism of action of CERS1 in NSCLC BM remains unclear. In this study, we elucidated for the first time that CERS1 partially suppressed NSCLC BM by downregulating the PI3K/AKT/mTOR signaling pathway. The PI3K signaling pathway is enriched in NSCLC BM and is associated with shorter BM-free survival [40]. Several activation-specific proteins of the PI3K/AKT pathway are highly expressed in BM compared to extracranial metastases [41]. Moreover, inhibition of the PI3K/AKT pathway may suppress BM occurrence in melanoma [42]. The association between CERS1 and the PI3K/AKT/mTOR signaling pathway in our study is a newly found and important supplement to the mechanism of CERS1 in malignant tumors. Nevertheless, whether CERS1 regulates BM through other signaling pathways warrants further research.

However, our study had some limitations. The sample size of BM tissues was relatively small and may have influenced the observed relationship between CERS1 and NSCLC BM. Furthermore, whether CERS1 regulates other molecules downstream of the PI3K/AKT/mTOR signaling pathway thus far remains unclear. Finally, in the future, an animal cancer model of NSCLC metastasizing to the brain should be used. 

## 5. Conclusions

In summary, we concluded that CERS1 plays a suppressor role in NSCLC BM by interacting with USP14 and downregulating the PI3K/AKT/mTOR signaling pathway, which may serve as a novel therapeutic target for NSCLC BM.

## Figures and Tables

**Table 1:** Correlation between the expression level of the CERS1 protein and clinical data of patients with BM.

Characteristic	CERS1		χ^2^	*p*-Value *
	Low Expression n = 21 (%)	High Expression n = 19 (%)		
Age (year)				
<60	9 (42.9)	11 (57.9)		
≥60	12 (57.1)	8 (42.1)	0.902	0.342
Median (range)	61 (35–75)	57 (44–75)		
Gender				
Male	9 (42.9)	9 (47.4)		
Female	12 (57.1)	10 (52.6)	0.082	0.775
Smoking statue				
No	14 (66.7)	11 (57.9)		
Yes	7 (33.3)	8 (42.1)	0.327	0.567
KPS score				
>80	7 (33.3)	10 (52.6)		
80	12 (57.1)	8 (42.1)		
<80	2 (9.6)	1 (5.3)	1.567	0.457
Pathological type				
Squamous cell carcinoma	4 (19.1)	3 (15.8)		
Adenocarcinoma	17 (80.9)	16 (84.2)	0.073	0.787
Differentiation				
High	2 (9.5)	8 (42.1)		
Median	10 (47.6)	9 (47.4)		
Low	9 (42.9)	2 (10.5)	8.027	0.018

* χ^2^ test was conducted to investigate the discrepancy in distribution between the two groups.

## Data Availability

The data that support the findings of this study are available upon request from the corresponding author. The data are not publicly available due to privacy or ethical restrictions.

## Supplementary Materials

The following supporting information can be downloaded at: https://www.mdpi.com/article/10.3390/cancers15071994/s1, Figure S1: CCLE dataset was used to analyze CERS1 expression in 28 malignant tumor cells; Figure S2: Effect of CERS1 on NSCLC cell migration and cell cycle; Figure S3: Verifying the permeability of the in vitro BBB model; Figure S4: Potential interacting proteins of CERS1 were identified via pull-down and LC-MS/MS analyses; Figure S5: Relevant key genes in apoptosis signaling pathways enriched by CERS1 overexpression in NSCLC. Table S1: The primer sequences used in this part of the experiment are as follows; Table S2: The specific shRNA sequence construction framework is as follows; Table S3: PCR amplification primers for overexpression CERS1 were showed in the following table.
