# Supplementary material for: Ceramide Synthase 1 Inhibits Brain Metastasis of Non-Small Cell Lung Cancer by Interacting with USP14 and Downregulating the PI3K/AKT/mTOR Signaling Pathway
Source: Cancers (Basel). 2023 Mar 27;15(7):1994. doi: 10.3390/cancers15071994 (PMC10093008; doi:10.3390/cancers15071994)
Supplement: Supplementary file 1 [file cancers-15-01994-s001.zip › cancers-2234878-supplementary.pdf]

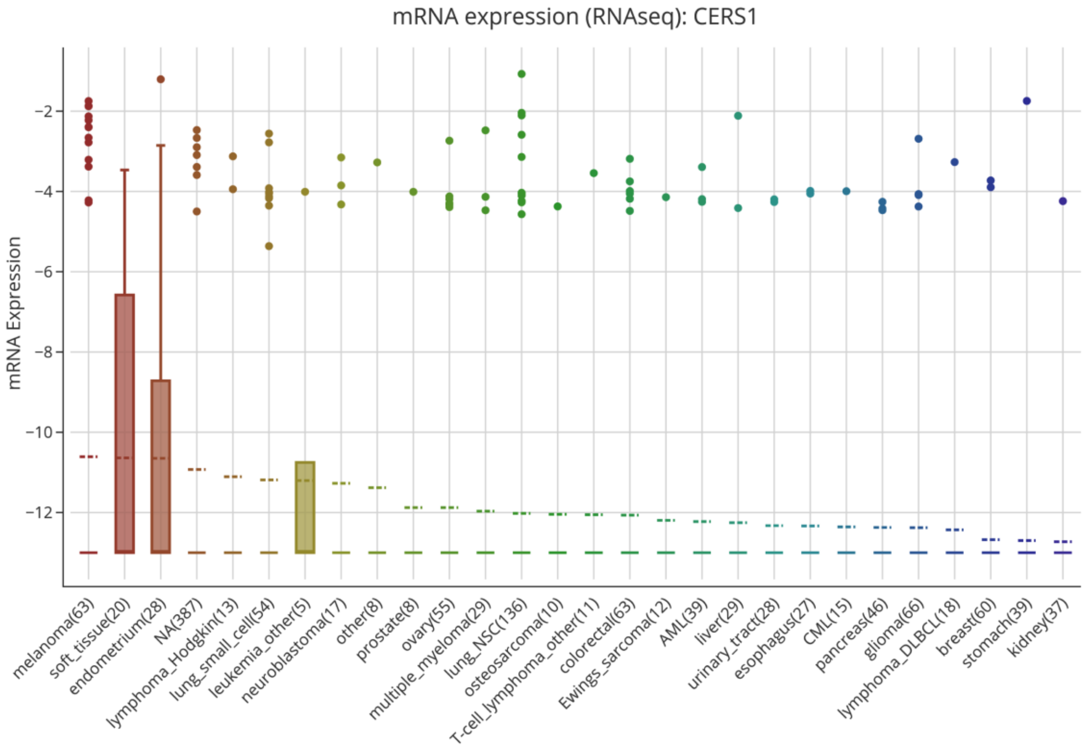

Figure S1. CCLE dataset was used to analyze CERS1 expression in 28 malignant tumor cells.

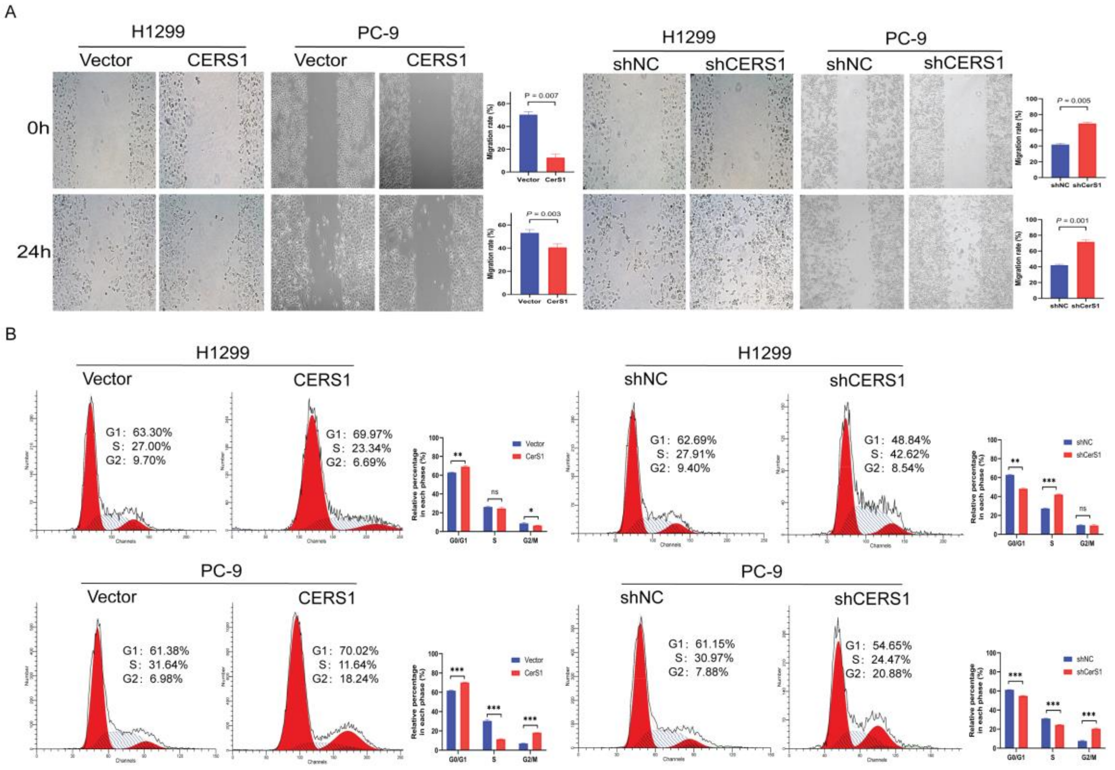

Figure S2. Effect of CERS1 on NSCLC cell migration and cell cycle. (A) Migration ability of cells following CERS1 transfection was tested via wound-healing assay. (B) Cell cycle was tested via flow cytometry after propidium iodide staining, and the data were analyzed using ModFit LT program.

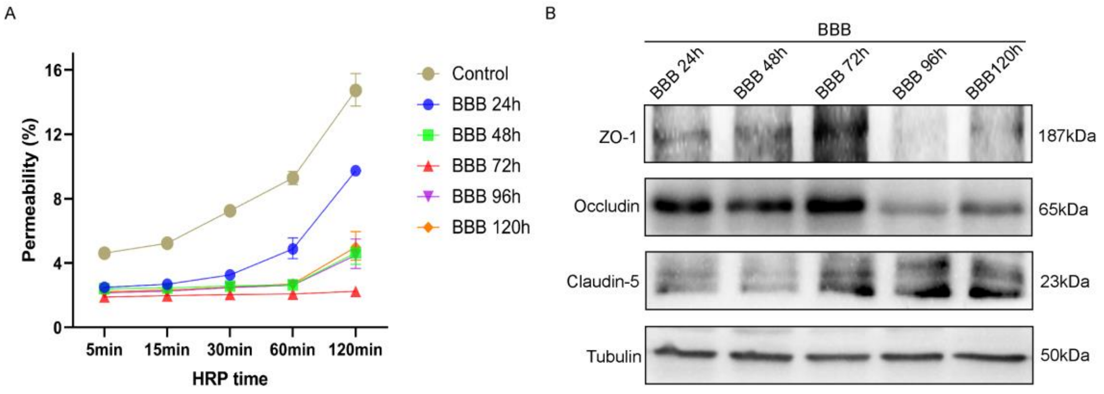

Figure S3. Verifying the permeability of the in vitro BBB model. (A) HRP flux was used to detect the permeability of BBB model. (B) The expression levels of tight junction-related proteins at the indicated times (24, 48, 72, 96, and 120 h) was performed to test the BBB permeability.

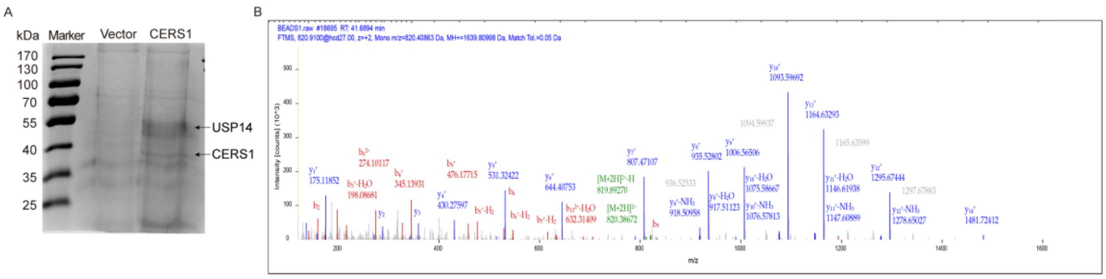

Figure S4. Potential interacting proteins of CERS1 were identified via pull-down and LC-MS/MS analyses. (A) Plot of pull-down. (B) Plot of secondary mass spectrometry.

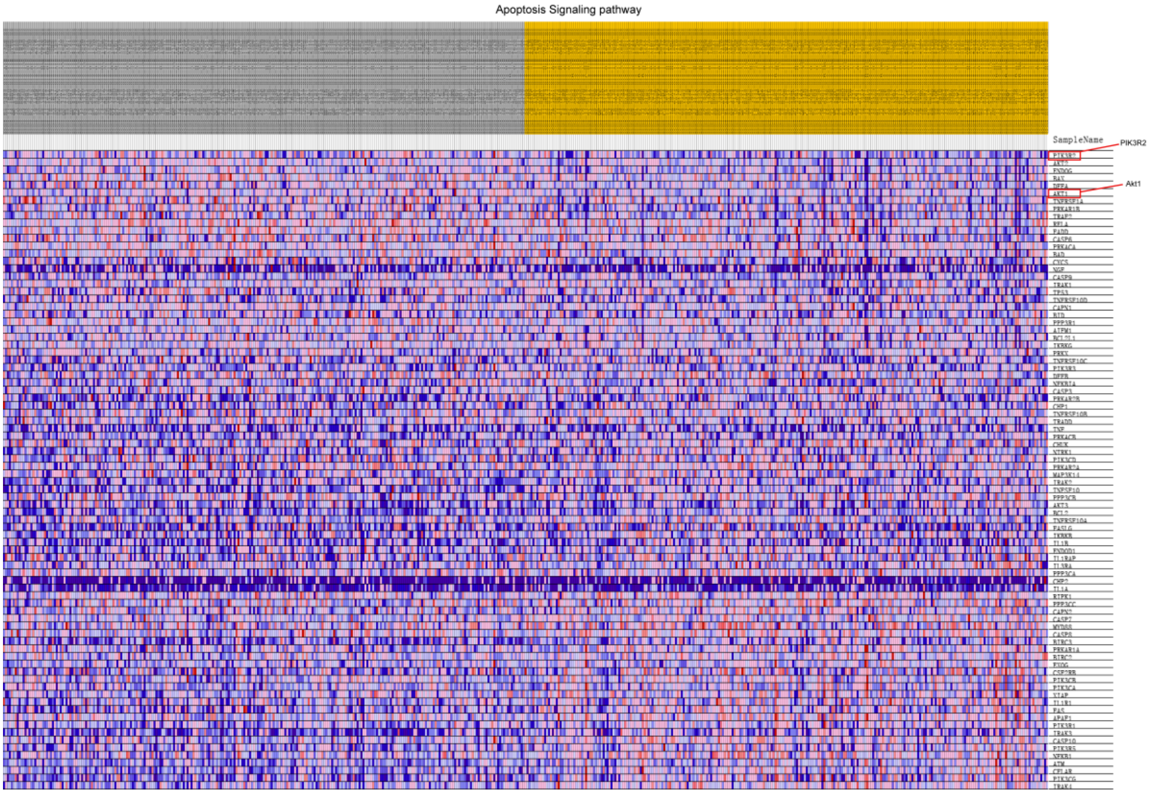

Figure S5. Relevant key genes in apoptosis signaling pathways enriched by CERS1 overexpression in NSCLC. Among them, PI3K and AKT were enriched in the apoptosis signaling pathway.

Table S1. The primer sequences used in this part of the experiment are as follows:.

Gene name	Primer sequence (5' to 3')
CERS1	Forward: CCTCCAGCCCAGAGAT<br>Reverse: AGAAGGGGTAGTCGGTG
CERS2	Forward: CCAGGTAGAGCGTTGGTT<br>Reverse: CCAGGGTTTATCCACAATGAC
CERS3	Forward: CCTGGCTGCTATTAGTCTGAT<br>Reverse: TCACGAGGGTCCCACT
CERS4	Forward: CTGGTGGTACCTCTTGGAGC<br>Reverse: CGTCGCACACTTGCTGATAC
CERS5	Forward: CAAGTATCAGCGGCTCTGT<br>Reverse: ATTATCTCCCAACTCTCAAAGA
CERS6	Forward: AAGCAACTGGACTGGGATGTT<br>Reverse: AATCTGACTCCGTAGGTAAATACA
GADPH	Forward: AATCCCATCACCATCTTCCAG<br>Reverse: GAGCCCCAGCCTTCTCCAT

Table S2. The specific shRNA sequence construction framework is as follows:.

Name		5’	STEMP	Loop	STEMP	3’
CERS1 #1	Forward	CCGG	GTGCTCTTCCTGCACGATATC	CTCGAG	GATATCGTGCAGGAAGAGCAC	TTTTTTG
	Reverse	AATTCAAAAAA	GTGCTCTTCCTGCACGATATC	CTCGAG	GATATCGTGCAGGAAGAGCAC	
CERS1 #2	Forward	CCGG	GCTGCTGCTCACCCTTATGAA	CTCGAG	TTCATAAGGGTGAGCAGCAGC	TTTTTTG
	Reverse	AATTCAAAAAA	GCTGCTGCTCACCCTTATGAA	CTCGAG	TTCATAAGGGTGAGCAGCAGC	
CERS1 #3	Forward	CCGG	GCTTCTATGGCCACTCCATCT	CTCGAG	AGATGGAGTGGCCATAGAAGC	TTTTTTG
	Reverse	AATTCAAAAAA	GCTTCTATGGCCACTCCATCT	CTCGAG	AGATGGAGTGGCCATAGAAGC	

Table S3. PCR amplification primers for overexpression CERS1 were showed in the following table:.

Id	Seq (5’→3’)
CERS1-Forward	GAGGATCCCCGGGTACCGGTCGCCACCATGCCCGAGAGCGCTTGGAAG
CERS1-Reverse	TCACCATGGTGGCGGGATCCAGCCCAGCAAAGCCGAGTGA
